# Exchange of the l-cysteine exporter after *in-vivo* metabolic control analysis improved the l-cysteine production process with engineered *Escherichia coli*

**DOI:** 10.1186/s12934-025-02715-y

**Published:** 2025-04-28

**Authors:** Daniel Alejandro Caballero Cerbon, Dirk Weuster-Botz

**Affiliations:** School of Engineering and Design, Chair of Biochemical Engineering, Technical University of Munich, Boltzmannstr. 15, 85748 Garching, Germany

**Keywords:** *E. coli*, l-cysteine, l-cysteine exporter, Metabolic control analysis, Fed-batch process, Dual substrate feeding

## Abstract

**Background:**

l-Cysteine is a proteinogenic amino acid of high pharmaceutical and industrial interest. However, the fermentation process for l-cysteine production is faced with multiple obstacles, like the toxicity of l-cysteine for the cells, the low carbon yield of the product, and the low selectivity of the l-cysteine exporter. In previous work, *in-vivo* metabolic control analysis (MCA) applied to an l-cysteine fed-batch production process with *E. coli,* followed by the targeted metabolic engineering to reduce an intracellular O-acetylserine (OAS) deficiency, resulted in a significant improvement of the l-cysteine production process with the new producer strain.

**Results:**

In this work, *in-vivo* MCA was applied to the l-cysteine fed-batch production process with the new producer strain (*E. coli* W3110 pCysK). The MCA indicated that a simultaneous increase in the exporter's expression and selectivity is required to increase the l-cysteine production further. The exchange of the l-cysteine exporter YdeD present in the plasmid pCysK for the potentially more selective exporter YfiK led to an increase of the maximal l-cysteine concentration by the end of the fed-batch process of 37% to a final concentration of 33.8 g L^−1^. The l-cysteine production could also be extended for at least 20 h due to conserved cellular activity as a result of the reduction of carbon loss as OAS.

**Conclusions:**

It could be shown that the *in-vivo* MCA methodology can be utilised iteratively with cells from the production process to pinpoint targets for further strain optimisation towards a significant increase in the l-cysteine production with *E. coli*. The use of this technology in combination with process engineering to adapt the fed-batch process to the modified strain may achieve a further improvement of the process performance.

**Supplementary Information:**

The online version contains supplementary material available at 10.1186/s12934-025-02715-y.

## Background

l-cysteine is a non-essential proteinogenic amino acid with a wide range of commercial applications. In the pharmaceutical industry, it acts as a precursor of N-acetyl-l-cysteine, which is in turn used in humans among several other applications to reduce oxidative stress, enhance glutathione production and reduce narcotics dependency [1]. In the food industry, l-cysteine enhances the dough quality of baked goods by breaking the disulfide bonds of proteins in the flour. It has been used in the cosmetic industry as an ingredient for permanent hair wave preparations. In animal feed, it covers the sulphur requirements of cattle and enhances aesthetic factors in wool production [2, 3].

Although the original l-cysteine production process involving the acidic hydrolysis of animal hair and feathers has been progressively phased out in favour of a biotechnological fermentation approach, some challenges in the microbiological l-cysteine production still need to be addressed [4]. These challenges include the high cytotoxicity of l-cysteine and its precursor O-acetylserine [5], the low carbon yield observed in the whole cell production [6], and the precursor OAS loss due to its co-export with the l-cysteine exporter [7, 8]. OAS reacts in the fermentation medium spontaneously to the isomer N-acetylserine (NAS), which then accumulates in the medium [9].

In our previous work, we applied a rational approach for strain engineering of *Escherichia coli* that increased the volumetric productivity of an l-cysteine fed-batch production process with dual feeding of glucose and thiosulfate (TSO) on a 15 L scale by 70% by increasing the expression of the enzyme l-cysteine synthase [10]. This strain engineering strategy was based on the results of an *in-vivo* Metabolic Control Analysis (MCA) performed with cells withdrawn from the fed-batch production process.

During the *in-vivo* MCA, short-term perturbation studies are performed with these cells within 21 min in parallel. The intracellular metabolite concentrations and flux distributions of the adjusted 12 metabolic steady states can be used to calculate elasticities, which are a measure of the effect of a change in the metabolite concentration in the activity of an enzyme [11]. These elasticities are, in turn, utilized to calculate flux control coefficients, which indicate the effect of a change in the concentration or activity of an enzyme over the metabolic flux through an enzymatic step inside a pathway [12]. The interpretation of these control coefficients allows the identification of enzymes that play a key role in a metabolic pathway, be it because they catalyse a rate-limiting step or because they facilitate the flux deviation towards an alternative pathway [13].

The application of *in-vivo* MCA on the l-cysteine production process with *E. coli* W3110 pCys resulted in the identification of two enzymes that catalysed the transformation of O-acetylserine into l-cysteine as rate-limiting for the overall l-cysteine biosynthesis pathway in the dual feeding fed-batch process. The overexpression of these enzymes led to significant increases in l-cysteine production, as evidenced by the 47% increase in the attained final l-cysteine concentration and 70% increase in maximal cell-specific productivity during the early fed-batch phase.

A secondary insight of this *in-vivo* MCA was that the intracellular scarcity of O-acetylserine, caused by the substantial export of this precursor by the overexpressed l-cysteine exporter, was limiting the l-cysteine production. Although the already performed strain engineering managed to canalise part of the intracellular O-acetylserine flux towards l-cysteine production, the l-cysteine production process with the modified strain still presented substantial extracellular NAS concentrations, which were even higher than the main product's concentration. In order to alleviate the intracellular precursor OAS loss, the exchange of the l-cysteine exporter for a more selective alternative was suggested.

The exporter YdeD, which is overexpressed in the strain *E. coli* W3110 pCys, was first described by Dassler et al. (2000) [7]. This research group found out that overexpressing this exporter native to *E. coli* in strain W3110 increased the l-cysteine production by a factor of 20 from 3 to 73 mg L^−1^. They also observed the accumulation of 147 mg L^−1^ of N-acetylserine as a byproduct (double the concentration as the main product) when the exporter was overexpressed. The use of alternative exporters like the antibiotic resistance factor *bcr* [14] or the l-cysteine exporter YfiK [8] have demonstrated comparable or even higher l-cysteine production than the exporter YdeD in shaking flask experiments and, in the case of the exporter YfiK, a reduced O-acetylserine export is also theorized. This work elaborates on the effects of exchanging the overexpression of the exporter YdeD for the overexpression of the exporter YfiK with putative higher l-cysteine selectivity on a fed-batch l-cysteine production process on a 15 L scale.

## Methods

### Strain development

Strain *E. coli* W3110 pCysK was chosen as the starting point for this work. This *E. coli* producer strain was developed as a result of the *in-vivo* MCA performed previously where it was also shown to possess the highest l-cysteine productivity of the strains in the study [10]. The plasmid pCysK includes genes coding for tetracycline resistance under the constitutive promotor *ptetR*, a feedback-insensitive version of phosphoglycerate dehydrogenase [15] under the constitutive promoter *pserA1,2*, a feedback-insensitive serine acetyltransferase [16] under the constitutive promoter *pcysE*, the l-cysteine exporter YdeD from *E. coli W3110* [7] under the promoter *pGAPDH*, and l-cysteine synthetase A [17] under the constitutive promoter *ppia*.

To generate a strain with an alternative l-cysteine exporter, the gene *yfiK,* coding for the l-cysteine exporter characterised by Franke et al. [8], was isolated from *E. coli's* genomic DNA by means of polymerase chain reaction (PCR) using the tailor-made primers (Primer 1 and 2) found in Table 1A in the supplementary information. The plasmid backbone was then generated by amplifying the plasmid pCysK, leaving out the gene *ydeD* by using primers 3 and 4 (in Table 1A of the supplementary information) for the PCR procedure. The *yfiK* insert and the plasmid backbone were then put together through Gibson assembly [18]. The resulting plasmid was named pCysK_yfik.

**Fig. 1:**
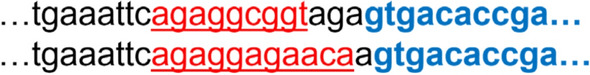
Engineering of the RBS for the exporter YfiK in the plasmid pCysK_yfiK. The upper sequence corresponds to the original RBS sequence native to the gene *ydeD* present in plasmid pCysK. The lower sequence corresponds to the modified RBS that closely resembles the native RBS of gene y*fiK.* In both cases, the RBS sequence has been underlined, and the start of the gene's coding region is marked with a bold script

To increase the expression level of the alternative l-cysteine exporter YfiK, the ribosome binding site adjacent to gene *yfiK* in plasmid pCysK_yfiK was engineered using the software UTR Designer [19] to achieve a comparable expression level as calculated for the exporter YdeD using the same software. The proposed new RBS sequence had an estimated expression level only 7.3% higher than that of the exporter YdeD with the original RBS. The changes in the RBS sequence were performed using a Q5 site-directed mutagenesis kit (E0554, New England Biolabs, Massachusetts, USA). The performed changes in the nucleotide sequence are presented in Fig. 1. The resulting plasmid was named pCysK_yfiK_nRBS.

The plasmid pCysK_yfiK_nRBS was transformed into chemically competent *E. coli* W3110 by means of heat shock transformation [20]. 10 µL of the plasmid solution with a concentration of 20.5 ng µL^−1^ were added to a frozen aliquot of 100 µL of competent *E. coli* W3110 cells on ice. The cells and plasmid solution were carefully mixed with the tip of a pipet, and the solution was left to rest for 30 min on ice. Subsequently, the reaction tube containing the transformation mixture was submerged in a water bath at 42 °C for 45 s. The reaction tube was then promptly put on ice and left there for 5 min. 500 µL of sterile LB medium [21] was added to the reaction tube, and the solution was incubated for 1 h at 37 °C. The solution was then plated into agar-LB plates (15 g L^−1^ agar–agar, 10 g L^−1^ peptone from casein, 5 g L^−1^ yeast extract, 5 g L^−1^ NaCl, autoclaved at 121 °C for 20 min, supplemented with 15 mg L^−1^ tetracycline hydrochloride and poured into sterile petri dishes) and incubated at 37 °C until colonies could be observed.

Cryo-cultures of the strain *E. coli* W3110 pCysK_yfiK_nRBS were generated by incubating the freshly transformed cells in a 100 mL shake flask without baffles containing 10 mL sterile lysogeny broth [21] supplemented with 15 mg L^−1^ tetracycline as selection marker overnight at 37 °C and 250 rpm in a shaking incubator (Infors HT, Bottmingen-Basel, Switzerland). The following day, 1 mL of the shake flask contents was transferred to a 250 mL shake flask without baffles containing fresh 24 mL lysogeny broth with antibiotic. After shaking for 4 h at 250 rpm and 37 °C, 8 mL of a sterile 60% glycerol solution was added to the shake flask. The resulting solution was aliquoted into sterile 2 mL plastic collection tubes that were instantly frozen by submersion into liquid nitrogen and were stored at − 80 °C until use.

The strain *E. coli* W3110 pCysK_yfiK_nRBS is available upon reasonable request via the corresponding author.

## Cultivation conditions

Unless otherwise specified, all media and equipment in this section were steam-autoclaved at 121 °C for 20 min. Stocks of temperature-sensitive components like the antibiotic, vitamins and amino acids were sterile-filtered and supplemented to the medium after autoclaving.

Culture expansion from cryo-culture stocks was performed in a two-stage process. First, 100 µL of the stock were used to inoculate six 1-L shake flasks with baffles containing 100 mL sterile lysogeny broth [21], each with 10 g L^−1^ d-glucose and 15 mg L^−1^ tetracycline. The flasks were shaken at 150 rpm and 37 °C for 8 h in the shake-flask incubator. The flasks' contents were centrifuged, and the supernatant was discarded. The pelleted cells were resuspended in sterile phosphate-buffered saline.

The resuspended cells were used to inoculate a stirred tank bioreactor with a working volume of 2 L (Labfors 3, Infors HT, Bottmingen, Switzerland). The utilized cultivation media was a modified version of the high cell density cultivation medium of Riesenberg et al. [22] consisting of 5 g L^−1^ (NH_4_)_2_SO_4_, 5 g L^−1^ KH_2_PO_4_, 1.2 g L^−1^ MgSO_4_·7H_2_O, 1 g L^−1^ trisodium citrate dihydrate, 0.5 g L^−1^ NaCl, 0.23 g L^−1^ CaCl_2_·2H_2_O, 75 mg L^−1^ FeSO_4_·7H_2_O, 1 g L^−1^ l-threonine, 0.9 g L^−1^ l-isoleucine, 0.6 g L^−1^ l-methionine, 90 mg L^−1^ pyridoxine, 18 mg L^−1^ thiamine and 10 mL L^−1^ of a trace element solution containing 3.75 g L^−1^ H_3_BO_3_, 1.55 g L^−1^ CoCl_2_·6H_2_O, 0.55 g L^−1^ CuSO_4_·5H_2_O, 3.55 g L^−1^ MnCl_2_·4H_2_O, 0.65 g L^−1^ ZnSO^4^·7H_2_O, and 0.33 g L^−1^ Na_2_MoO_4_·H_2_O. As a carbon source, 40 g L^−1^ d-glucose was supplied to the reactor before inoculation. The temperature was controlled to 37 °C through a heat exchange jacket. The pH was kept constant at pH 7 by titration with a solution of 25% ammonia or 6.8 N H_3_PO_4_ as required. The oxygen supply was performed by dispersion of pressurised air from the bottom of the reactor at a rate of 2 vvm. The dissolved oxygen concentration was kept over 40% air saturation by regulating the stirrer speed between 600 and 1200 rpm (two 6-bladed Rushton impellers). The cells were cultivated overnight until they reached an optical density measured at 600 nm (OD_600_) of 30.

Afterwards, the reactor content was transferred to a stainless-steel 42 L stirred tank bioreactor (Techfors HT, Infors, Bottmingen, Switzerland) containing 10 L of the minimal medium described above. The stirred tank bioreactor counts with two feeding inlets, three titration solution inlets, a sterile, pressurised air inlet, a sampling port, a safety valve calibrated at 3 bar, a heat-exchange jacket for steam and cooling water, a bottom-up stirrer with three 6-bladed Rushton impellers of 10 cm diameter, and sensors for pH, temperature, vessel pressure and dissolved oxygen concentration. Temperature and pH conditions were kept constant throughout the process at 32 °C and pH 7 through the same procedures described for the 2 L stirred tank bioreactor. The dissolved oxygen (DO) concentration was controlled to 40% air saturation by regulation of the stirrer speed. Pressurised air was supplied to the bioreactor at a rate of 2 vvm, and an absolute pressure of 1.7 bar was applied in the vessel.

As a batch carbon source, 10 g L^−1^ d-glucose were supplied. Once the batch glucose was wholly consumed and the DO signal increased over 60% air saturation, the feeding with 670 g L^−1^ d-glucose was automatically started, marking the beginning of the fed-batch phase. Two hours after inoculation, a feeding with 320 g L^−1^ ammonium thiosulfate was started in order to provide the cells with the necessary sulphur for l-cysteine production. The exact profiles of the feeding streams are provided in Fig. A1 in the supplementary information.

For both the 2 L and the 42 L stirred tank bioreactors, the online data was gathered using the process manager software IRIS V5.3 (Infors HT, Bottmingen-Basel, Switzerland).

Sampling was performed during the day at 1.5 h intervals through the sampling port at the bottom of the reactor. The medium samples were frozen at − 20 °C and thawed again shortly before their analysis.

## Parallel short-term experiments

During the point in time in the fed-batch l-cysteine production process, when the l-cysteine productivity reached a maximal value, which remained constant throughout a time frame of several hours, a 4 L sample was rapidly withdrawn from the reactor. After withdrawal from the reactor, the cell solution undergoes an established rapid media transition protocol [23].

The rapid media transition protocol consisted of transferring the 4 L sample to four 1 L centrifuge containers, taring the containers and centrifuging at 3200 *g* for 10 min in a centrifuge tempered at 32 °C (Rotixa 500 RS, Andreas Hettich GmbH, Tuttlingen, Germany). The supernatant is discarded, and the cells are resuspended in 400 mL fresh minimal medium tempered at 32 °C. The resuspended cell solution is aliquoted in four 250 mL graduated cylinders, so that each of the graduated cylinders contains approximately 100 mL. The cylinders’ contents are used for the inoculation of four parallel stirred tank reactors (fourfold Dasgip® Bioblock-System, Eppendorf SE, Hamburg, Germany) with a working volume of 0.5 L of minimal medium each.

The medium contained 9 g L^−1^ ammonium thiosulfate to ensure the preservation of the l-cysteine production. To keep the experiment conditions similar to the reference process in the 42 L scale, the temperature was set at 32 °C and pH 7 was kept constant through the addition of either 2 M NaOH or 21% H_3_PO_4_. Aeration was provided at a rate of 8 vvm and consisted of a mixture of pressurised air and pure oxygen, yielding an inlet oxygen volumetric fraction of 25%. Stirring, performed by two six-bladed Rushton impellers in each reactor, was kept constant at 1200 rpm. Online process data was gathered from the process manager software Dasgip Control (Eppendorf SE, Hamburg, Germany). The oxygen and carbon dioxide contents of the exhaust gas from the parallel stirred tank bioreactors were measured by an at-line gas analyser (EL2030, ABB Germany, Mannheim, Germany).

In order to generate perturbations of the metabolism of the cells from the reference state in the 42 L bioreactor, the parallel bioreactors were each fed with a different carbon source. Solutions of either 35 g L^−1^ d-glucose, 52 g L^−1^ pyruvate, a glucose (39 g L^−1^)/pyruvate (32 g L^−1^) mixture, and a glucose (28 g L^−1^)/succinate (18 g L^−1^) mixture were selected as carbon sources. These carbon sources were selected because of their distinct points of entry into the central carbon metabolism (glucose as a starting point of the glycolysis, pyruvate favoring the gluconeogenetic pathways and succinate supplementing the metabolic flow in the citrate cycle part of the citrate cycle) which have shown to be effective in generating metabolic perturbations in previous studies with short-term experiments [10, 24–26]. Each of the carbon sources was fed at three different constant feeding rates (30, 60, and 90 ml h^−1^), with each feeding stage lasting 7 min so that the metabolism of the *E. coli* cells could reach a metabolic steady state [24].

To generate metabolome and fluxome databases of the metabolism perturbations for the metabolic analysis, samples were withdrawn from each of the parallel bioreactors at the beginning and the end of each of the feeding stages as well as from the 42 L l-cysteine production process that was used as reference state. The samples taken at the end of each stage were, additionally, instantaneously sprayed into a metabolism inactivation solution at − 54 °C consisting of 40% methanol and 60% 30 mM triethanolamine via a tailor-made rapid sampling and quenching system [27].

A U-^13^C-labelled cell extract was used as an internal standard for intracellular metabolite determination. This cell extract was prepared by cultivating *E. coli* W3110 pCysK cells in the adapted mineral medium described above for the fed-batch l-cysteine production process. As glucose source, U-13C d-glucose was supplied. The cells were cultivated in batch operation in the presence of 4 g L^−1^ thiosulfate to encourage the l-cysteine biosynthetic path. The cells were then concentrated by centrifugation at 3000 *g* and 37 °C for 10 min and resuspended in phosphate-buffered saline. The labelled intracellular metabolites were extracted by aliquoting the cell solution in a 1:10 ratio with 30 mM triethanolamine solution at 95 °C and heating at the same temperature for 5 min.

The intracellular metabolites of the inactivated samples from the short-term experiments were extracted by the addition of 300 µL of the sample and 300 µL of the ^13^C internal standard into 2.4 mL of a 30 mM triethanolamine solution at 95 °C and heating at this temperature for 5 min.

## Analytical methods

Two milliliter of each of the thawed samples from the 42 L reactor and the parallel experiments were aliquoted into a 2 mL collection tube, which was then centrifuged at 23,000 *g* and 20 °C for 10 min (Centrifuge 5424 R, Eppendorf SE, Hamburg, Germany). The supernatant was separated (supernatant 1) for HPLC analysis. To resuspend the precipitated l-cystine, 1 mL of an acidic mixture consisting of 12% (v/v) H_3_PO_4_ and 1.5% (v/v) H_2_SO_4_ was added to the collection tube containing the centrifuged pellet. The tube was closed and vigorously shaken on a vortexer for 10 min. The tube was then centrifuged again at 23,000 *g* and 20 °C for 10 min. The supernatant was collected for HPLC determination (supernatant 2), and the cell pellet remaining in the collection tube was dried in a drying oven at 80 °C for 48 h before gravimetrical cell dry weight determination. The cell dry weight was calculated as the difference in weight between the empty centrifuge tube after being brought to constant weight by placing it in the drying oven at 80 °C for 48 h and the weight of the tube with the cell pellet after drying. Dividing the resulting value over the sample volume of 2 mL yields the biomass concentration for each sample.

The glucose, phosphate, malate, pyruvate, succinate, formate, acetate, and ethanol determinations were carried out via HPLC analysis. 20 µL of the supernatant 1 samples were injected in a cation exchange chromatography column (Aminex HPX-87H, Bio-Rad Laboratories, California, USA) with an isocratic eluent flow of 0.7 mL min^−1^ phosphoric acid 5mM. The analytes were detected by a refractive index detector (1100 series, Agilent, California, USA).

Thiosulfate, l-cysteine, and N-acetylserine were quantified via HPLC by injecting 2 µL of supernatant 1 into a reverse-phase chromatography column (Gemini 5 µm C18 110Å 250 × 4.6 mm, Phenomenex, California, USA) with an isocratic flow of 0.5 ml min^−1^ of an acidic mixture composed of 5.4 mM H_2_SO_4_ and 58.6 mM H_3_PO_4_. Detection was performed by a UV/Vis detector at 200 nm (DAD-3000, Thermo Fisher Scientific, Waltham, USA). The column was washed after each sample for 5 min with a flow of 1 ml min^−1^ of acetonitrile. The quantification of the precipitated l-cystine concentration was performed with the same HPLC method, injecting 2 µL of supernatant 2.

The quantification of intracellular metabolites from the extracted short-term experiment samples was performed via the Liquid Chromatography—Mass Spectrometry (LC–MS) methodology for the quantification of metabolic intermediates described by Buescher et al. [28].

The LC–MS system (Thermo Fisher Scientific, Waltham, USA) consisted of an autosampler (Thermo Pal), a piston pump (Accela), a column oven (MistraSwitch) and a triple quadrupole detector (TSQ Vantage), all connected to the device control and data acquisition and interpretation software Thermo Xcalibur (Thermo Fisher Scientific, Waltham, USA). 20 µl of the extracted samples from the short-term experiments were injected into a C18 reverse-phase UHPLC separation column (Acquity HSS T3, Waters Corporation, Milford, USA), which was heated at 40 °C. The eluent profile consisted of a 36-min gradient from 100% solvent A (15 mM acetic acid, 10 mM tributylamine and 5% methanol) to 100% solvent B (100% Isopropanol). The analytes were ionised by an electro-sprayer with a voltage of 2.8 kV, a capillary temperature of 380 °C, and a vaporiser temperature of 400 °C. The metabolites were identified by a triple quadrupole detector on its negative polarity operation. The intracellular metabolite concentrations of the ^13^C-labelled cell extract used as internal standard were measured as well. The ratio of the intracellular metabolite concentrations of the untreated ^13^C extract and the ^13^C metabolite concentrations of the internal standard in the extracted short-term experiment samples was used as a correction factor to account for thermal metabolite degradation during the extraction.

## Metabolic analysis

The flux balance analysis (FBA) and flux variance analysis (FVA) were carried out using the Python package of the COBRA program for quantitative prediction of metabolic flow distributions [29]. The *E. coli* genome-scale metabolic model iJO1366 [30] was used as the base for the metabolic flux analysis. The missing reactions from the enzymes sulfo-l-cysteine synthase, sulfo-l-cysteine lyase, sulphite reductase, and thiosulfate transferase, as well as the metabolite sulfo-l-cysteine, were added to the model. The thermodynamic flux balance analysis (TFA) was performed using the Python package pyTFA [31]. For this, the identifiers, molecular weight, pKa, structure cues, and ΔG_f_ of the metabolite S-sulfo-l-cysteine had to be supplied to the model. The ΔG_f_ value was estimated based on the group contribution methodology of Mavrovouniotis [32].

The extracellular fluxes of substrate uptake, product formation, and gas exchange determined during the short-term experiments were used in the FBA to calculate intracellular flux distributions. Subsequently, a loopless FVA was carried out to eliminate biologically unfeasible metabolic flux loops and to take into account experimental error during the extracellular flux determinations by relaxing the threshold of the flux distributions from the FBA to all the flux distributions that reached 99.9% of the optimal value of the objective function (growth rate) achieved by the FBA solution.

The solution space generated by the FVA was further constrained during the TFA by the addition of thermodynamical constraints that forced the reactions in the most thermodynamically feasible direction. The solution space consisted of ranges for every Gibbs reaction energy, reaction rate, and metabolite concentration in the model, which could then be transferred to the reduced metabolic model for the MCA. This model consisted of 37 reactions and 40 metabolites from the glycolysis, pentose phosphate, citrate cycle, and l-cysteine synthesis pathways.

The MCA was programmed and performed on Matlab R2021a (Mathworks, Natick, USA). The model's elasticities were calculated using either the thermokinetic affinity model for reactions near thermodynamic equilibrium [33, 34] or the lin-log approach [11] for reactions far from thermodynamic equilibrium. The concentration control coefficients and flux control coefficients were calculated according to the equations developed by Visser and Heijnen [35]. To observe the stability of the computed control coefficients towards inaccuracies in the experimental measurements of metabolite concentrations and extracellular fluxes, the MCA was carried out within the framework of a Monte Carlo sampling algorithm with 10,000 iterations according to the protocol established by Wang et al. [36].

## Results

### *In-vivo* MCA with E. coli W3110 pCysK withdrawn from the fed-batch process

**Fig. 2:**
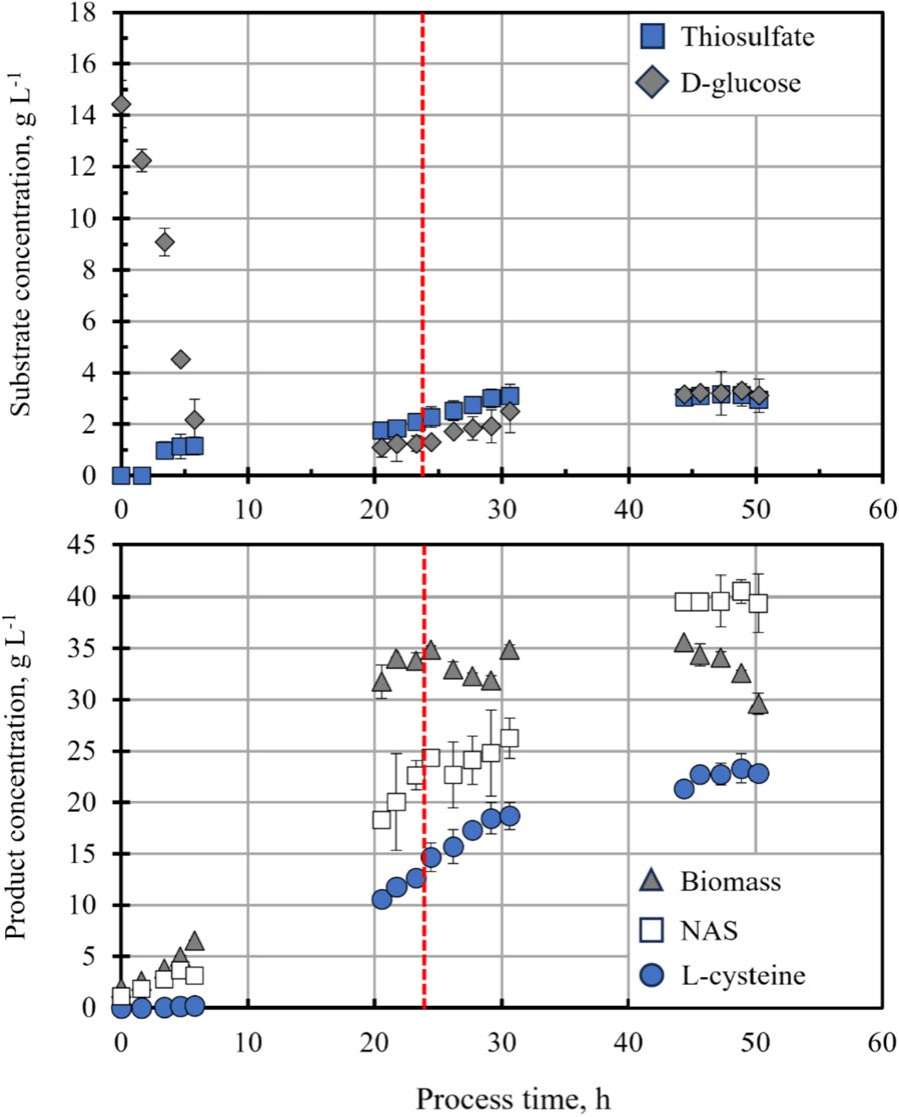
Fed-batch l-cysteine production with *E. coli* W3110 pCysK on a 15-L scale. Top: d-glucose (*diamonds*) and thiosulfate (*squares*) concentrations. The feeding profiles of both substrates are shown in the supplemental information of this work. Bottom: Biomass (*triangles*), l-cysteine (*circles*), and NAS (*squares*) concentrations. The stirred tank bioreactor was operated at 32 °C, 1.7 bar, pH 7, aeration of 20 NL min^−1^ sterile, pressurized air, stirrer speed 300–1000 rpm and DO > 40% air saturation. The vertical dotted line indicates the point in time when 4 L of the reactor content were withdrawn for the short-term experiments. The error bars indicate the standard deviation of the three technical replicates for each sample

A fed-batch l-cysteine production process with *E. coli* W3110 pCysK was carried out on a 15 L scale to serve as a reference process for further metabolic analysis. The concentration profiles in the cultivation media for the supplied substrates and obtained products of this process are shown in Fig. 2. The reproducibility of this process has been demonstrated in our previous work [10].

The process was inoculated with enough cells from the preculturing to reach a biomass concentration of 1.8 g L^−1^. Shortly after inoculation, the cells grew exponentially with a maximal growth rate of 0.21 h^−1^ while they consumed the 10 g L^−1^ of glucose initially present in the medium. Seven hours after inoculation, the initial glucose was completely depleted, and the fed-batch operation of the bioreactor was automatically started. Twenty hours after inoculation, the culture reached a stationary phase, where the biomass concentration increased no further and reached a maximal value of 35.6 g L^−1^. Throughout the fed-batch operation, the glucose concentration in the medium remained between 1.1 and 3.3 g L^−1^, indicating that the feeding strategy was adequate and that no carbon supply limitation took place.

The biomass concentration decreased gradually 44 h after inoculation, reaching a final biomass concentration of 29.6 g L^−1^ within 50 h after inoculation when the process was terminated. The decline of the biomass concentration towards the end of the cultivation can be linked to a decrease in cellular activity, which was observed for this process in the gas exchange rates calculated from the exhaust gas data of the stirred tank bioreactor (Fig. A2 in the Supplementary Information of this publication).

The thiosulfate concentration followed a similar profile to the glucose concentration since the feeding rates of both substrates were chosen to prevent substantial accumulation in the medium. From the start of the thiosulfate supply, the thiosulfate concentration in the medium was kept between 1.0 and 3.2 g L^−1^.

The l-cysteine production commenced between 7 and 20 h after inoculation and presented a constant productivity of 995.2 mg L^−1^ h^−1^ in a timeframe between 20 and 30 h after inoculation. During this period of constant maximal l-cysteine production, 23 h after inoculation, the sampling for the short-term experiments was performed. Thirty hours after inoculation, the l-cysteine productivity decreased in parallel with the cell activity. A maximal l-cysteine concentration of 23.3 g L^−1^ was observed towards the end of the process.

Besides l-cysteine, the exporter YdeD also exports the precursor O-acetylserine (OAS) from the cells into the medium, where it reacts into N-acetylserine (NAS) and constitutes the main byproduct of the process. The overproduction of this precursor is linked to the initial strain design that enabled the production of l-cysteine. Even with the strain improvement performed in our previous work, NAS still accumulates in the medium at concentrations that far surpass the main product, l-cysteine. The constitutive expression of the genes responsible for the OAS overproduction and its export from the cells causes an accumulation of NAS in the medium from the beginning of the cultivation. In this process, a maximal concentration of NAS of 40.5 g L^−1^ was recorded by the end of the cultivation.

**Fig. 3:**
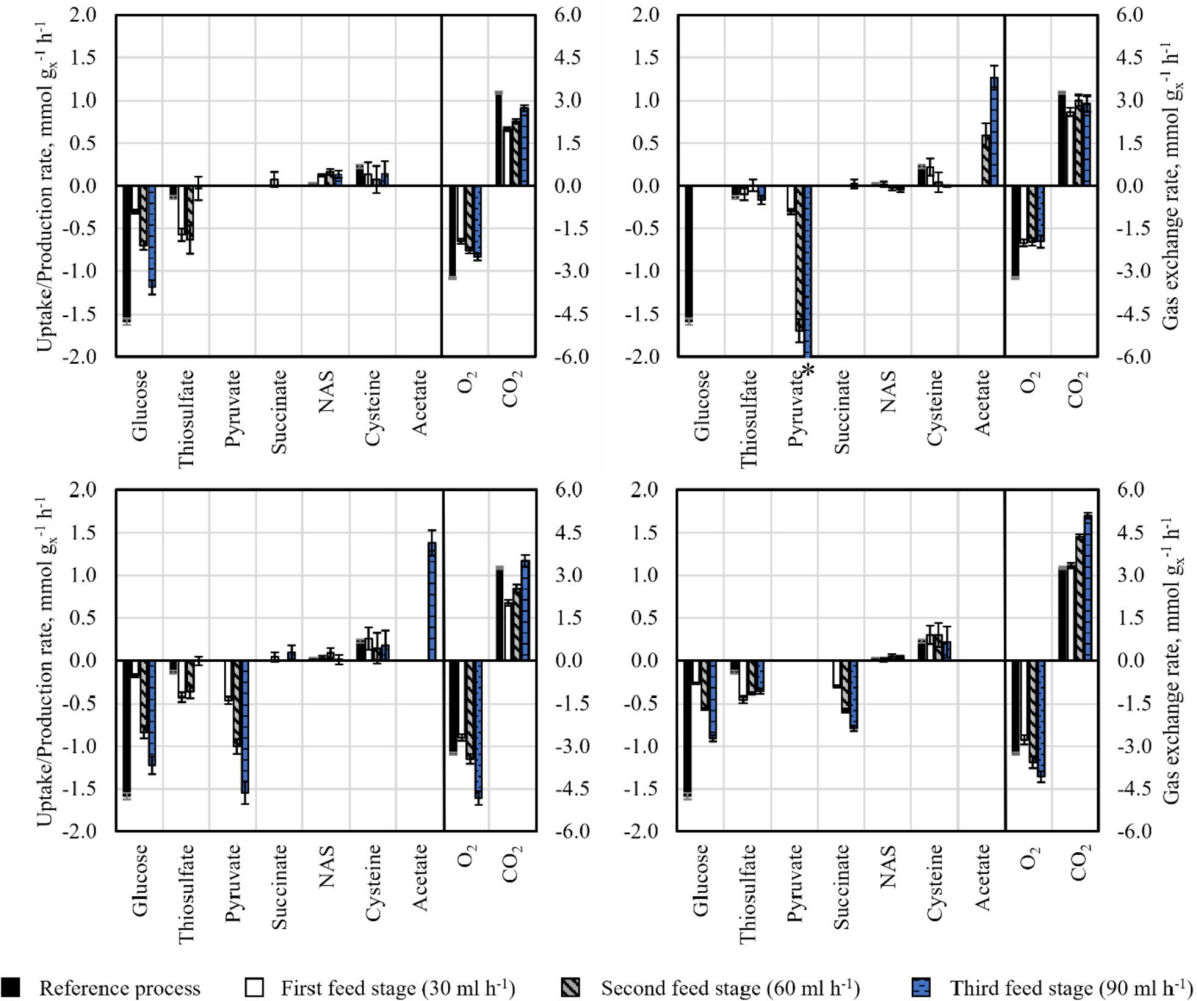
Extracellular rates derived from the short-term perturbation experiments performed with *E. coli* W3110 pCysK. Top left: Parallel reactor fed with glucose as sole carbon source. Top right: The parallel reactor fed with pyruvate as sole carbon source. Bottom left: The parallel reactor fed with a mixture of glucose and pyruvate. Bottom right: The parallel reactor fed with a mixture of glucose and succinate. The reference state (black bars) is presented in each graph for comparison. The rates of the first (30 ml h^−1^—white bars), second (60 ml h^−1^—grey bars) and third (90 ml h^−1^—blue bars) feeding stages are presented side by side for each reactor. Negative rates indicate uptake, whereas positive rates indicate production. The parallel stirred-tank reactors were operated at 32 °C, pH 7, 1 bar, aeration with a mixture of pure oxygen and air to a final oxygen fraction in the inlet of 24% v/v (* − 2.2 mmol g_X_^−1^ h^−1^)

The cells withdrawn from the 15 L bioreactor sampling 23 h after inoculation underwent a rapid media transition protocol and were used to inoculate the four parallel stirred tank bioreactors on a 0.5 L scale, where short-term perturbation studies were carried out. Each of the parallel reactors was inoculated with an initial biomass concentration of 28.3 g L^−1^. The estimated extracellular rates for the four parallel bioreactors are summarised in Fig. 3.

It is possible to observe three distinct increasing substrate uptake rates for each of the 4 carbon sources utilised to perturbate the metabolism of the cells from its original state in the reference 15 L scale process. Each of these three substrate rates corresponds to one of the three feeding stages of 30, 60, and 90 ml h^−1^ of the concentrated stock solution of carbon source. Similar increasing behaviour with increasing feeding rate can be observed in the gas exchange rates: the oxygen uptake rate and the carbon evolution rate. The sole exception is the last stage of the reactor with pyruvate as a single carbon source, where the increased pyruvate influx was diverted to enhanced acetate production. It is essential for metabolic analyses that the generated steady states still show l-cysteine production since only then are the calculated metabolic distributions relevant to the optimisation of the l-cysteine production process. In these short-term experiments, an l-cysteine production rate higher than 0 g L^−1^ h^−1^ was observed for all perturbation states. In total, 12 distinct metabolic equilibrium states were generated, which, together with their corresponding measured intracellular metabolite concentrations, were used as input for the MCA.

The metabolome (Table A2 in the supplementary information) and extracellular rates data from the 12 equilibrium stages reached in the short-term experiments and from the reference process were used for the FBA, FVA, and TFA to calculate 13 different intracellular carbon flux distributions, which are summarised in Table A1 of the supporting information for selected reactions of the central carbon metabolism, pentose phosphate pathway, citrate cycle, and l-cysteine biosynthesis pathway.

The TFA also provided information on the Gibb's free energy of reaction for all the reactions in the model according to the calculated carbon fluxes and the intracellular metabolite concentrations. This information is necessary for the calculation of elasticities during the MCA since the method of elasticity calculation is dependent on how far each reaction in the model is from thermodynamical equilibrium. Reactions whose Gibb's free energy of reaction was between 0 and − 10 kJ mol^−1^ were considered to be near thermodynamical equilibrium. On the other hand, reactions with a Gibb's free energy of reaction below − 10 kJ mol^−1^ were considered far from equilibrium [37].

The reactions in the reduced metabolic model for the MCA (Table 1) that were found to be near thermodynamic equilibrium were glucose 6-phosphate isomerase (PGI), fructose bisphosphatase A (FBA), triose-phosphate isomerase (TPI), glyceraldehyde 3-phosphate dehydrogenase (GAPD), phosphoglycerate kinase (PGK), phosphoglycerate mutase (PGM), enolase (ENO), aconitase (ACONT), succinyl-coA synthase (SUCOAS), fumarase (FUM), malate dehydrogenase (MDH), ribose phosphate epimerase (RPE), ribose-5-phosphate isomerase (RPI), transketolase 1 (TKT1), transaldolase (TALA), phosphoglycerate dehydrogenase (PGCD), phosphoserine transaminase (PSERT).

**Table 1:** Reduced metabolic model for MCA

ID	Reaction
PTS	Glucose (ext) + PEP → G6P + Pyr
PGI	G6P → F6P
PFK	F6P + ATP → FDP + ADP
FBA	FDP → DHAP + GAP
TPI	DHAP → GAP
GAPD	GAP + NAD + P_i_ → 13DPG + NADH
PGK	13DPG + ADP → 3PG + ATP
PGM	3PG → 2PG
ENO	2PG → PEP + H_2_O
PDH	NAD + Pyr + CoA → AcCoA + NADH + CO_2_
PPC	PEP + CO_2_ + H_2_O → OAA + P_i_
ACS	Acetate + ATP + CoA → AcCoA + AMP + PPi
CS	AcCoA + OAA + H_2_O → Citrate + CoA
ACONT	Citrate → Isocitrate
ICDH	Isocitrate + NADP → AKG + NADPH + CO_2_
AKGDH	AKG + CoA + NAD → SucCoA + NADH + CO_2_
SUCOAS	SucCoA + ADP + P_i_ → Succinate + ATP + CoA
SUCDH	Succinate + Q8 → Fumarate + Q8H_2_
FUM	Fumarate + H_2_O → Malate
MDH	Malate + NAD → OAA + NADH
G6PDH	G6P + NAD → 6PGC + NADH
GND	6PGC + NADP → Ru5P + NADPH + CO_2_
RPE	Ru5P → Xu5P
RPI	Ru5P → R5P
TKT1	Xu5P + R5P → GAP + S7P
TKT2	Xu5P + E4P → F6P + GAP
TALA	GAP + S7P → F6P + E4P
ACS	ATP + Acetate + CoA → AMP + AcCoA + PP_i_
PGCD	NAD + 3PG → NADH + 3PHP
PSERT	3PHP + L-glutamate → Pser + AKG
PSPL	Pser + H_2_O → P_i_ + L-ser
SERAT	AcCoA + L-ser → OAS + CoA
CYSS	OAS + H_2_S → lcys + Acetate
SLCYSS	OAS + S_2_O_3_^2−^ → S-Sulfocysteine + Acetate
SCYSSL	S-Sulfocysteine + NADPH → l-cys + SO_3_^2−^ + NADP
YdeD	OAS + l-cys → OAS (ext) + l-cys (ext)
NADH5	NADH + Q8 → NAD + Q8H_2_
ATPS	ADP + Pi → ATP

The enzymatic steps and metabolites are labelled in accordance with their identifier in the BiGG Models databank [38], which was used as a basis for the construction of the genome-wide and the reduced model

The reactions of the glucose import through the pyruvate transfer system (PTS), phosphofructokinase (PFK), pyruvate dehydrogenase (PDH), phosphoenolpyruvate carboxylase (PPC), acetyl-coA synthetase (ACS), citrate synthase (CS), isocitrate dehydrogenase (ICDH), α-ketoglutarate dehydrogenase (AKGDH), succinate dehydrogenase (SUCD), glucose-6-phosphate dehydrogenase (G6PDH), phosphogluconate dehydrogenase (GND), transketolase 2 (TKT2), phosphoserine phosphatase (PSPL), serine acetyltransferase (SERAT), l-cysteine synthase (CYSS), S-sulfo-l-cysteine synthase (SLCYSS), S-sulfo-l-cysteine lyase (SCYSSL), l-cysteine and O-acetylserine export (YdeD), NADH dehydrogenase (NADH5) and ATP synthase (ATPS) were considered to be far from thermodynamic equilibrium.

**Fig. 4:**
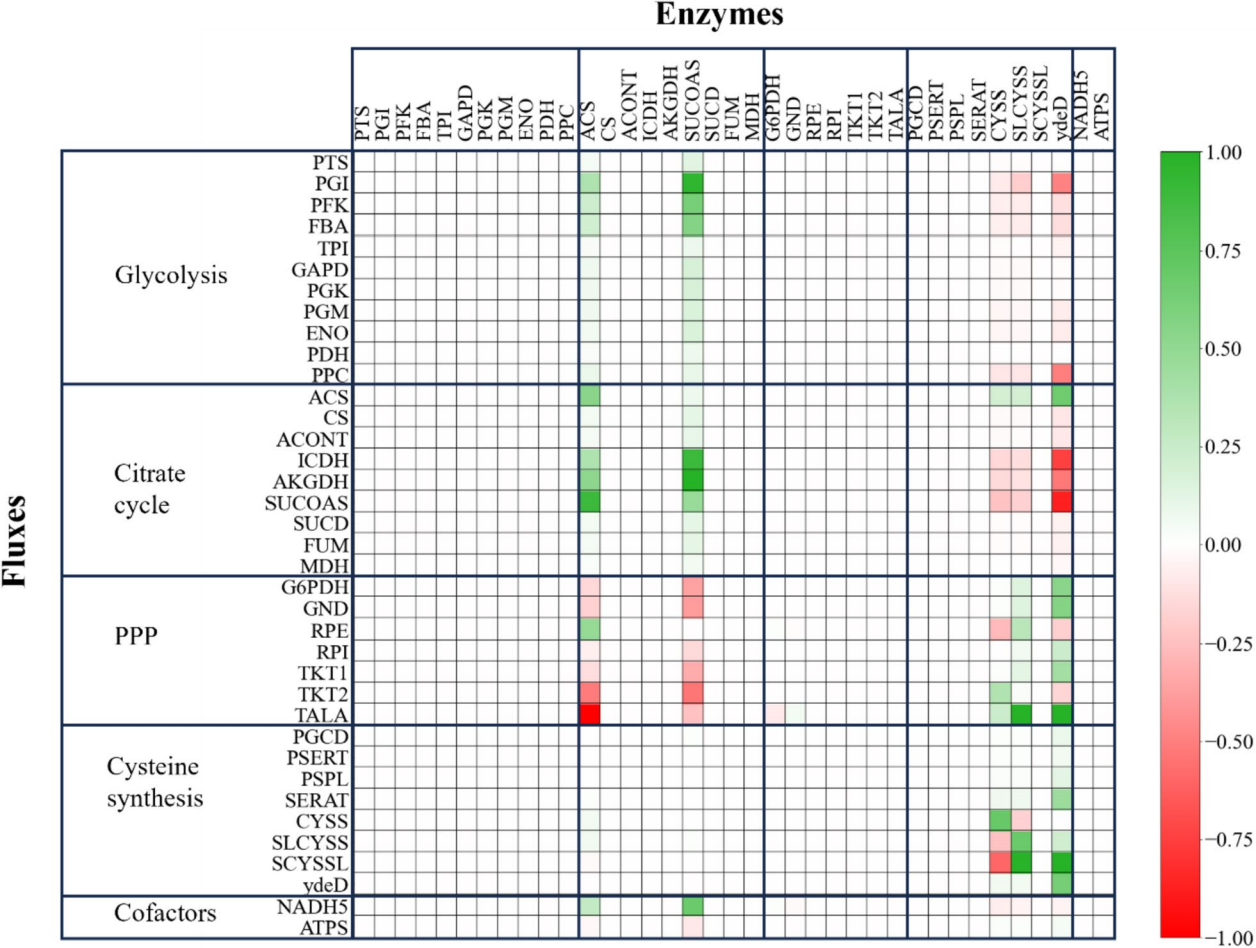
Flux control coefficients derived by *in-vivo* MCA with cells withdrawn from the fed-batch l-cysteine production process with *E. coli* W3110 pCysK. The colour indicates the percentual effect that a 1% change in the concentration of the enzymes in the columns has over the metabolic fluxes through the enzymatic steps in the rows. A positive value indicates that an increase in the activity or concentration of the enzyme in the column would lead to a higher flux through the reaction in the respective row and vice versa

The calculated flux distributions and metabolite concentrations were utilised during the MCA to determine the control relationship between metabolite and enzyme concentrations and the metabolic fluxes through specific pathways. The effect of metabolite concentrations on the activity of a particular enzyme is characterised by the so-called elasticities. Together with the stoichiometric model and the flux distributions, the elasticities are used in the MCA for the calculation of control coefficients. The final result of the MCA is the flux control coefficients, which quantify the effect of a percentual change in the activity or concentration of an enzyme on the metabolic flux through an enzymatic step [13]. A summary of the flux control coefficients calculated for the l-cysteine-producing strain *E. coli* W3110 pCysK can be observed in Fig. 4.

The high degree of control that both the acetyl CoA synthase (ACS) and the succinyl CoA synthase (SUCOAS) exert over the central carbon metabolism may be explained by their effect on the citrate cycle. ACS facilitate the carbon transition from the glycolysis to the citrate cycle by transforming a byproduct of the glycolysis (acetate) into the starting point of the cycle, namely acetyl CoA. On the other hand, SUCOAS, as written in the model, catalyses the transformation of succinyl CoA into succinate. SUCOAS may be a rate-limiting step of the citrate cycle since an increase in this enzyme's concentration is favourable for the flux through their two preceding steps, as indicated by the flux control coefficients of ICDH and AKGDH. SUCOAS also provides the glycolysis with ATP, which explains the positive control coefficients observed in the metabolic fluxes through PGI, PFK, and FBA since the middle reaction PFK requires ATP to transform fructose-6-phosphate into fructose bisphosphate.

The negative control coefficients in the pentose phosphate pathway for the enzymes ACS and SUCOAS can be explained by the positive effect of both enzymes in the competing pathway in the glycolysis for the utilization of fructose-6-phosphate and the alternative production of reduction equivalents in the enhanced citrate cycle that reduces the need for the reduction equivalents from the first two reactions (G6PDH and GND) of the pentose phosphate pathway.

Concerning the l-cysteine biosynthesis pathway, the highest degree of control of the metabolic flux towards the l-cysteine production is localised in three enzymes: CYSS, SLCYSS, and the exporter YdeD. It is possible to observe a competitive relationship between the enzymes CYSS and SLCYSS, which was also reported in our previous work. This competitive relationship is characterised by negative flux control coefficients for the enzymes towards the metabolic flux through the opposing enzyme and positive control coefficients towards the flux of their own enzymatic step. This is caused by the export of OAS from the microbial cell, which serves as a precursor for both competing enzymes. The competitive relationship between CYSS and SLCYSS persists despite the strain engineering (overexpression of CYSS) performed after the previous MCA because the main reason for the intracellular OAS scarcity, namely the OAS export, was not addressed. The overexpression of CYSS only improved the utilisation of the limited intracellular OAS before it could be exported. The control coefficients of the flux through SCYSSL are an enlarged reflection of the control coefficients for the flux through SLCYSS because the rate of reaction of the former enzyme is entirely dependent on the production of S-sulfo-l-cysteine catalyzed by SLCYSS.

Even though the export of OAS by the exporter YdeD is the main cause of intracellular OAS deficiency, the exporter had a positive flux control coefficient over most fluxes of the l-cysteine biosynthesis pathway, especially over SCYSSL, the last enzyme in the l-cysteine production path from thiosulfate. This suggests that the benefits of a faster l-cysteine export outweigh the carbon loss as OAS for a net gain in flux towards the l-cysteine production if the exporter activity is further increased.

The negative control coefficients of the enzymes CYSS, SLCYSS and the exporter YdeD over the metabolic fluxes through the glycolysis can be explained by the deviation of the carbon flux from the glycolysis into the l-cysteine biosynthetic pathway, which the overexpression of these proteins may cause. The sole exception is the metabolic flux through ACS since the l-cysteine biosynthetic pathway produces acetate as a byproduct, which is a substrate of the reaction catalysed by ACS.

## l-cysteine production with* E. col*i W3110 pCysK_yfiK_nRBS

The results of the *in-vivo* MCA performed on the l-cysteine production process with *E. coli* W3110 pCysK imply that increasing the exporter concentration while simultaneously addressing the reason for the intracellular OAS scarcity would lead to a higher metabolic flux towards the l-cysteine production.

The expression of the exporter YdeD is already regulated by a strong constitutive promoter in the plasmid pCysK. Additionally, a further increase in the *ydeD* expression would possibly exacerbate the OAS scarcity in the cells. For these reasons, an alternative exporter with a higher selectivity towards l-cysteine was sought. The l-cysteine exporter YfiK fo *E. coli* was characterized by Franke et al. [8], who theorised that this exporter would provide higher selectivity towards l-cysteine over OAS when compared to YdeD. However, no data was presented to corroborate this statement.

**Fig. 5:**
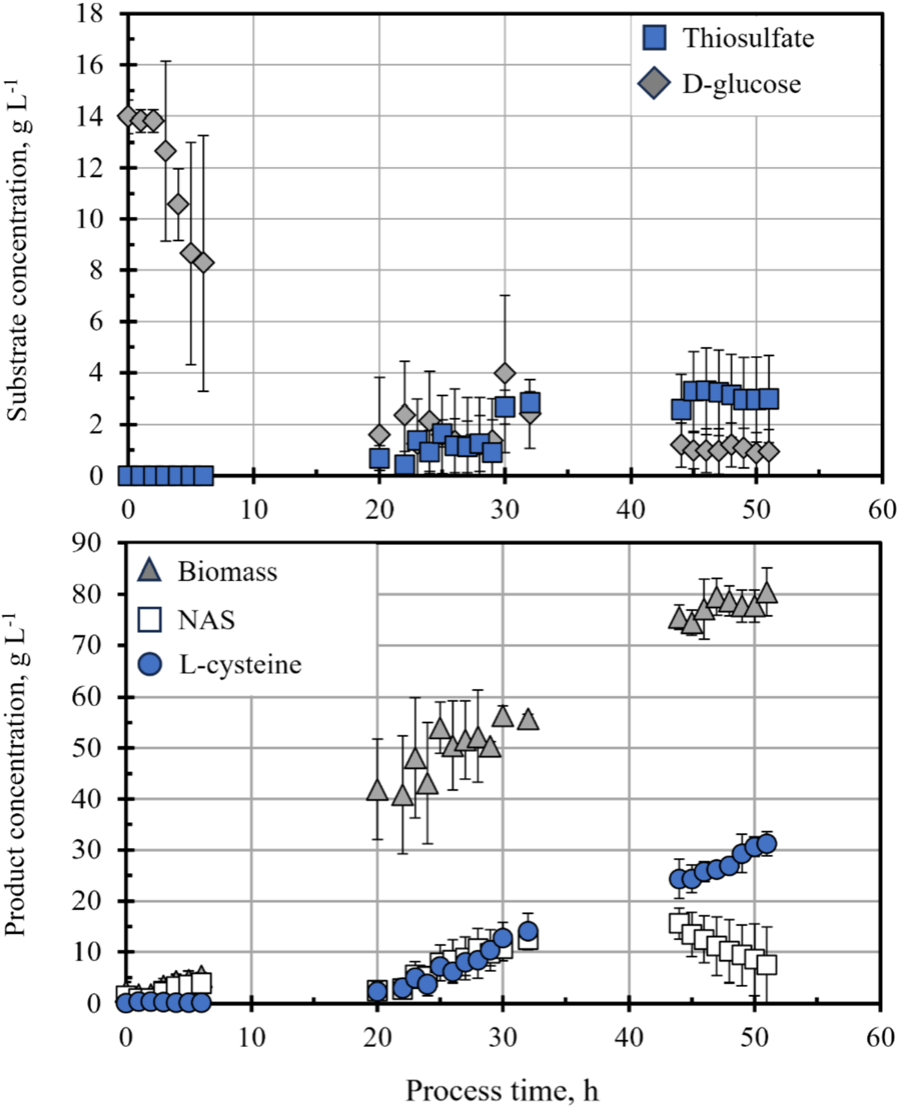
Fed-batch l-cysteine production process with *E. coli* W3110 pCysK_yfiK_nRBS on a 15 L-scale. **a** Glucose (*diamonds*) and thiosulfate (*squares*) concentration profiles. **b** Biomass (*triangles*), NAS (*squares*) and l-cysteine (*circles*) concentration profiles. The stirred tank bioreactor was operated at 32 °C, 1.7 bar, pH 7, aeration of 20 NL min^−1^ sterile, pressurised air, stirrer speeds of 300–1000 rpm, and DO > 40% air saturation. The error bars indicate the standard deviation of the concentration profiles of three independent l-cysteine production processes

The sequence for the overexpression of *ydeD* was exchanged by the corresponding sequence of the exporter YfiK from *E.* coli W3110 in the plasmid pCysK to generate plasmid pCysK_yfiK_nRBS. The fed-batch l-cysteine production process with the strain *E. coli* W3110 pCysK_yfiK_nRBS was carried out in triplicate to characterise the effect of this exporter change in the l-cysteine production. The average substrate and product concentration profiles of these three production processes with this strain are presented in Fig. 5.

For ease of comparison, the glucose and thiosulfate feeding profiles for the fed-batch operation of the l-cysteine production process were kept identical to the ones utilised for the fed-batch l-cysteine production process with *E. coli* W3110 pCysK. Just as in the process with the exporter YdeD, the substrate supply rates were adequate to prevent substantial substrate accumulation. However, glucose limitation during the beginning of the fed-batch operation cannot be ruled out as the glucose concentration between 18 and 24 h after inoculation is merely 0.2 g L^−1^. The reason for this glucose shortage in the medium is the improved biomass growth.

In this fed-batch process, the exponential biomass growth phase gave way to a face of reduced growth during the fed-batch operation instead of the stationary phase observed in the l-cysteine production process with the strain carrying the plasmid pCysK. This additional growth phase allowed the biomass to reach a final concentration of 85 g L^−1^ by the end of the process 51 h after inoculation. This increased biomass formation is most likely a result of the reduced carbon loss as OAS since the maximal NAS concentration in the medium of the fed-batch process with *E. coli* W3110 harbouring the plasmid pCysK_yfiK_nRBS was decreased to 19.9 g L^−1^. This result demonstrates that the OAS export of the *E. coli* cells was cut down by half by the exchange of l-cysteine exporters.

The reduction of the OAS export did not have an adverse effect on l-cysteine production. On the contrary, there was an uninterrupted l-cysteine accumulation until the end of the process and a maximal concentration of 33.8 g L^−1^ was reached 51 h after inoculation. A maximal volumetric productivity of 2.052 g L^−1^ h^−1^ was observed for this process between 41 and 51 h after inoculation.

The reduction of the OAS export led to a maintained cellular activity, as observed by the increase in the biomass and l-cysteine concentrations even towards the end of the cultivation and by the gas exchange rates, which even increased slightly towards the end of the process (as shown in Fig. A3 in the supplementary information). The fed-batch process was stopped 51 h after inoculation because the objective was to compare it to the l-cysteine production process with the strain carrying the plasmid pCysK, which by this time had already reached a constant l-cysteine concentration. However, the increasing tendency of the l-cysteine concentration means the l-cysteine production phase could be extended further to increase the final l-cysteine concentration.

The increase in selectivity by the exporter exchange was quantified by comparison of the cell-specific export rates of OAS and l-cysteine for the fed-batch processes with each of the two exporters. In the process with the exporter YdeD carried out in triplicate the average export rate of l-cysteine during the maximal productivity stage between 20 and 30 h after inoculation was 24.6 mg g^−1^ h^−1^, whereas the export rate of OAS for the same time period was 30.1 mg g^−1^ h^−1^, yielding a selectivity (the ratio of desired product production rate to by-product production rate) of 0.81. On the other hand, the average l-cysteine export rate of the l-cysteine production process with exporter YfiK between 20 and 30 h of cultivation was 20.7 mg g^−1^ h^−1^ (the smaller cell-specific rate in comparison to the process with YdeD being a result of an increased biomass concentration) while the export rate of OAS for this point in time being 17.6 mg g^−1^ h^−1^ resulting in an significantly increased l-cysteine selectivity of 1.76.

**Fig. 6:**
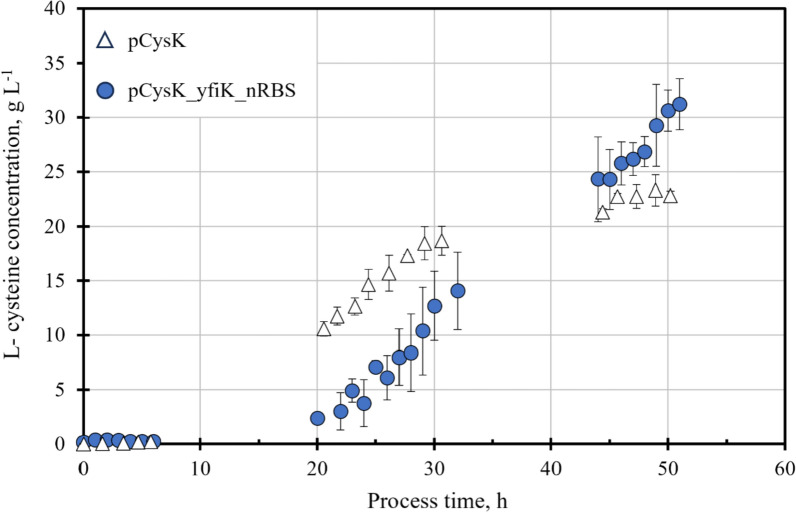
Comparison of the l-cysteine concentration profiles of the fed-batch l-cysteine production processes on a 15 L scale with *E coli* W3110 carrying plasmid pCysK (*triangles*) or plasmid pCysK_yfiK_nRBS (*circles*). Each of the profiles was generated by averaging three fed-batch processes with the same strain. The error bars indicate the standard deviation between data points of three independent runs of the fed-batch l-cysteine production process. The stirred tank bioreactor with an initial volume of 10 L was operated at 32 °C, pH 7, 1.7 bar, aeration with 20 NL min^−1^ sterile air, DO > 40% air saturation, and stirred speeds of 300–1000 rpm

To facilitate the comparison of both standardized fed-batch production processes with *E. coli* W3110 harbouring the plasmids with the l-cysteine exporter YdeD or YfiK, the l-cysteine concentration profiles are presented in Fig. 6. The maximal l-cysteine concentration by the end of the fed-batch process could be increased by 37% by expressing the more selective l-cysteine exporter YfiK. A single-tailed Student's *t*-test [39] performed over the three biological replicates of the fed-batch l-cysteine production process with the strains carrying each of the two exporters indicated that the final l-cysteine concentration for the processes with the exporter YfiK is significantly (*p* < 0.05) higher than that of the l-cysteine production process with the strain overexpressing the YdeD exporter. The delayed onset of the l-cysteine production in the process with the *E. coli* strain carrying the plasmid pCysK_yfiK_nRBS may be attributed to the prolonged biomass growth phase as a result of the increased cellular activity. Since the l-cysteine biosynthesis and central carbon metabolism must compete for the intracellular carbon flux, the majority of the l-cysteine is produced once the biomass growth slows down.

The maximal volumetric l-cysteine productivity registered for both processes is comparable, with 1.23 g L^−1^ h^−1^ from the process with the strain carrying the plasmid pCysK being slightly higher than the 1.08 g L^−1^ h^−1^ achieved by the process with the plasmid pCysK_yfiK_nRBS. This is most likely caused by the distribution of carbon between cell growth and product formation. The continuous biomass growth throughout the process with exporter YfiK most likely deviated carbon from the l-cysteine production leading to smaller l-cysteine productivities in the period between 20 and 30 h after inoculation in comparison with the process with exporter YdeD. However, due to this extended cellular activity of the strain carrying the exporter YfiK as a result of the decreased OAS export, the maximal volumetric productivity could be maintained over 20 h longer than in the process with the exporter YdeD.

A further extension of the fed-batch l-cysteine production process with *E. coli* W3110 pCysK_yfiK_nRBS to up to 77 h after inoculation, shown in Fig. A4 of the supplemental information, did not lead to significantly enhanced l-cysteine concentrations, since the l-cysteine productivity was observed to decrease rapidly after 50 h of cultivation. A slight increase in the l-cysteine concentration from 32.0 to 36.6 g L^−1^ was achieved by extending the fed-batch production process by 27 h. However, this change in the l-cysteine concentration falls within the standard deviation of the l-cysteine measurements for the samples in this period.

**Fig. 7:**
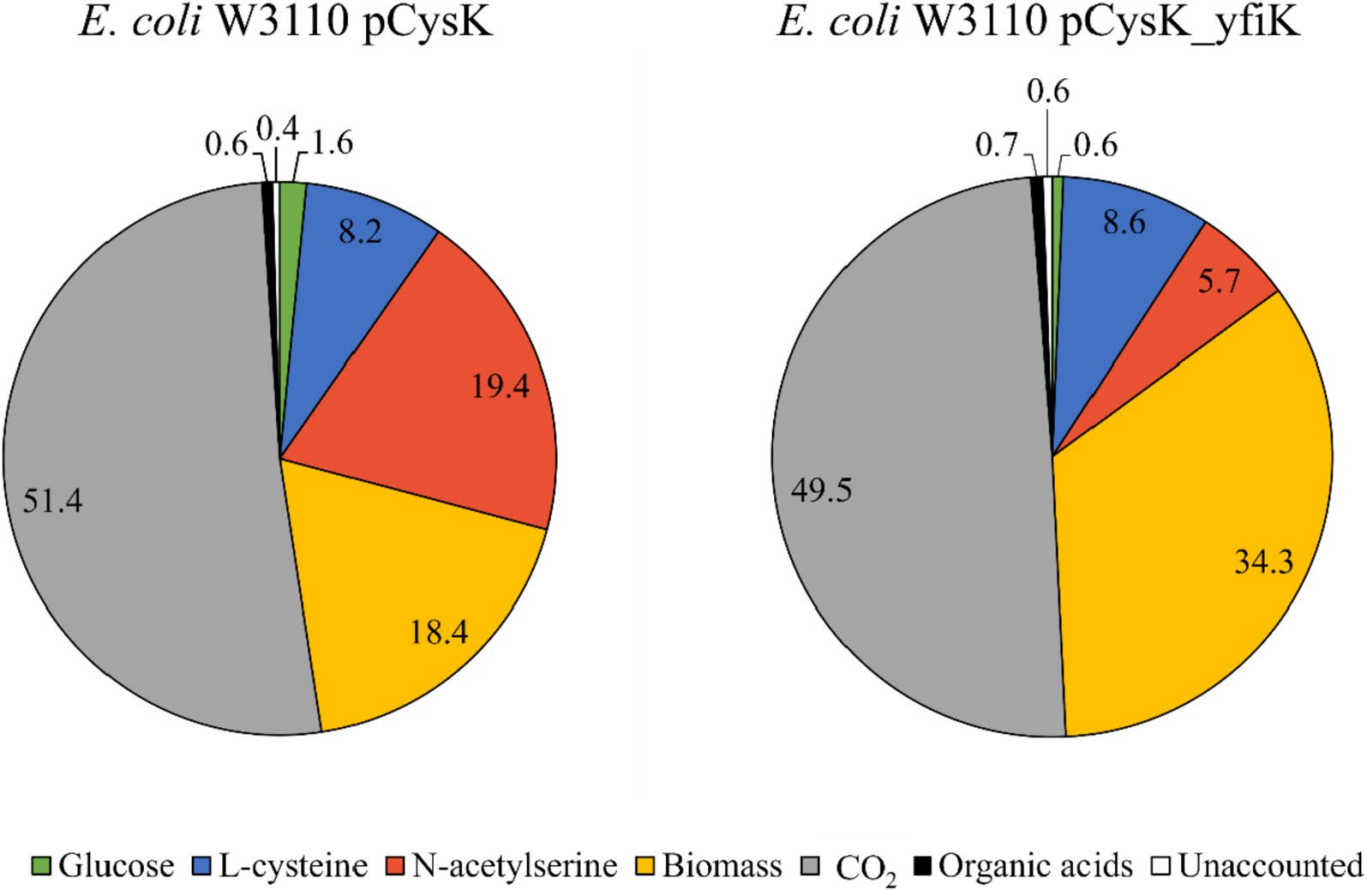
Carbon molar balances for the fed-batch l-cysteine production processes with *E. coli* W3110 pCysK (left) and *E. coli* W3110 pCysK_yfiK_nRBS (right). The shares presented in the pie diagrams correspond to the percentage of carbon moles supplied to the process in the form of d-glucose that could be found in the form of the shown respective components according to the analytical methods used in this work. The unaccounted fraction is the molar percentage necessary to close the carbon balances to 100%

The carbon molar balances for the fed-batch l-cysteine production process with strain W3110 pCysK and with strain W3110 pCysK_yfiK_nRBS are presented in Fig. 7. The completeness of the analytical determinations used in this work could be confirmed since, in both cases, more than 99% of the carbon entering the system as d-glucose could be accounted for via HPLC or exhaust gas analytics.

The clearest difference in the carbon utilization of these two processes lies in the NAS share. From the 19.4% of total carbon in the process with strain W3110 pCysK, the NAS was greatly reduced to only 5.7% in the process with strain W3110 pCysK_yfiK_nRBS. Most of the saved carbon went into additional biomass formation, which went from 18.4 to 34.3% with the exporter exchange. The l-cysteine share increased only 0.4% when the exporter YfiK was overexpressed instead of the exporter YdeD, meaning the saved carbon flux was rather canalized into the central carbon metabolism than into the l-cysteine biosynthesis. A further *in-vivo* MCA is likely to show increased flux control coefficients in the central carbon metabolism, affecting the flow towards the l-cysteine biosynthesis now that the primary source of intracellular O-acetylserine scarcity has been alleviated, thereby releasing the control from the immediate vicinity of the biosynthesis pathway. Flux control coefficients that deviate the carbon flux away from the CO_2_ production would also be of particular interest since, in both of these processes, around half of the carbon supplied to the reactor ends up being released as CO_2_.

## Conclusions

The *in-vivo* MCA of the l-cysteine production with *E. coli* W3110 pCysK indicated that an increase in the exporter activity coupled with a decrease in the OAS export would lead to a higher l-cysteine productivity. The exchange of the l-cysteine and OAS exporter YdeD with the exporter YfiK, which potentially possesses a higher selectivity towards l-cysteine against OAS, led to increased cellular activity, possibly as a result of the conserved carbon flux not being exported as OAS being diverted towards biomass growth and l-cysteine production. As a result, the l-cysteine production could be extended for at least 20 h in the process with the overexpression of the exporter YfiK in comparison with the process with the overexpression of the exporter YdeD. A significant increase in the final l-cysteine concentration of 37% was achieved through this exporter exchange.

The effectivity of the *in-vivo* MCA methodology for identifying targets for rational strain optimisation was demonstrated for the l-cysteine production process with *E. coli*. The iterative nature of this methodology could also be corroborated by the further increase in product concentrations, as it has been done before for repeated MCA and strain optimisation cycles in the aromatic amino acid production [25, 26, 40].

It is likely that by addressing the intracellular OAS deficiency, which has been a pervasive source of control over the l-cysteine biosynthetic pathway through two rounds of strain optimisation, the dynamics of flux control within the l-cysteine pathway have shifted. Since every metabolic control analysis can be considered an instant shot of the metabolic state of the cells at the time of the metabolome and transcriptome sample [10], new control coefficients for the carbon fluxes through the l-cysteine synthesis path which have not been significant in previous MCAs might be observed if an MCA over the l-cysteine production process with *E. coli* W3110 pCysK_yfiK_nRBS is performed. This especially holds true for the enzymes in the central carbon metabolism, which was greatly favoured by the exporter exchange canalizing the carbon previously lost as NAS to biomass generation.

From the results of the further improved strain in this work, it becomes clear that strain engineering must be coupled with process engineering to accommodate the novel needs of the modified strain. For this work in particular, the process conditions were kept constant throughout processes with different strains to facilitate the comparison of the l-cysteine productivities based solely on the utilised strain. However, in the fed-batch l-cysteine production process with *E. coli* W3110 pCysK_yfiK_nRBS, the substrate feeding rates must be modified to prevent substrate limitation, as was the case for glucose early in the fed-batch phase. Moreover, if the objective of a further study is not to compare the l-cysteine production capabilities of two strains within a strain optimization pipeline to characterize the effectiveness of the strain engineering methodology, as is the case in this work, but to maximise the final l-cysteine concentration, the process time of the fed-batch process with the strain overexpressing the exporter YfiK could be extended over the shown 51 h after inoculation with an optimised feeding strategy.

Furthermore, if the goal of further process optimization lies in industrial scalability, one of the first aspects to be taken into consideration is the high oxygen demand made evident by the elevated gas exchange rates of both l-cysteine production processes shown in the supplementary information, which may lead to oxygen deprivation in larger reactors due to the formation of local zones of low oxygen concentration as a consequence of longer mixing times with increasing reactor size. These heterogeneous cultivation conditions may lead to the formation of bacterial subpopulations with reduced cellular activity and productivity [41, 42].

## Supplementary Information

Additional file 1

## Data Availability

The datasets generated and analyzed during the current study are available from the corresponding author upon reasonable request.

## Abbreviations

MCA: Metabolic control analysis
TFA: Thermodynamic flux analysis
OAS: O-acetylserine
NAS: N-acetylserine
LC-MS: Liquid chromathography and mass spectrometry
HPLC: High-performance liquid chromatography
DO: Dissolved oxygen concentration
PGI: Glucose 6-phosphate isomerase
FBA: Fructose bisphosphatase A
TPI: Triose-phosphate isomerase
GAPD: Gglyceraldehyde 3-phosphate dehydrogenase
PGM: Phosphoglycerate mutase
ENO: Enolase
ACONT: Aconitase
FUM: Fumarase
MDH: Malate dehydrogenase
RPI: Ribose-5-phosphate isomerase
TALA: Transaldolase
PGCD: Phosphoglycerate dehydrogenase
PSERT: Phosphoserine transaminase
ATPS: Adenosine triphosphate synthase
PTS: Glucose transport via the pyruvate transfer system
PFK: Phosphofructokinase
PSP: Phosphoserine phosphatase
PDH: Pyruvate dehydrogenase
PPC: Phosphoenolpyruvate carboxylase
CS: Citrate synthase
ICDH: Isocytrate dehydrogenase
AKGDH: α-Ketoglutarate dehydrogenase
SUCCOAS: Succinyl-coA synthetase
SUCDH: Succinat dehydrogenase
G6PDH: Glucose-6-phosphate dehydrogenase
GND: Phosphogluconate dehydrogenase
RPE: Ribulose 5-phosphate 3-epimerase
TKT1: Transketolase1
TKT2: Transketolase 2
SERAT: Serine acetyltransferase
CYSS: l-cysteine synthase
SLCYSS: Sulfo-l-cysteine synthase
SCYSSL: Sulfo-l-cysteine lyase
NADH5: NADH dehydrogenase
PPP: Pentose phosphate pathway
