# Supplementary material for: Exchange of the l-cysteine exporter after in-vivo metabolic control analysis improved the l-cysteine production process with engineered Escherichia coli
Source: Microb Cell Fact. 2025 Apr 28;24:95. doi: 10.1186/s12934-025-02715-y (PMC12038998; doi:10.1186/s12934-025-02715-y)
Supplement: Supplementary file 1 — Additional file 1 [file 12934_2025_2715_MOESM1_ESM.pdf]

Supplementary information

Exchange of the L-cysteine exporter after metabolic control analysis improved L-cysteine production with engineered *Escherichia coli*.

Daniel Alejandro Caballero Cerbon (ORCID: 0009-0005-8681-6506), Dirk Weuster-Botz (ORCID: 0000-0002-1171-4194)

Technical University of Munich, School of Engineering and Design, Chair of Biochemical Engineering, Boltzmannstr. 15, 85748 Garching, Germany

(Corresponding author: dirk.weuster-botz@tum.de)

Table A1. Plasmids and primers used in this study. The plasmids are presented along with their overexpressed genes and respective promoters. The presented primers were utilised to exchange the exporter ydeD for the exporter yfiK in the plasmid pCysK to generate plasmid pCysK_yfiK

**Plasmids**	
**Name**	**Content**
**pCysK**	P_{tetR}-*TCR* $P_{serA1,2}$-*serA* P_{cysE}-*cysE* P_{fic}-*cysK* P_{GAPDH}-*ydeD*
**pCysK_yfiK**	P_{tetR}-*TCR* $P_{serA1,2}$-*serA* P_{cysE}-*cysE* P_{fic}-*cysK* P_{GAPDH}-*yfiK*
**Primers**	
**Name**	**Sequence**
**1. yfiK_fwd**	CGACATCTCGGGGCTTATTATTAATAGAAAATGCGTACCGC
**2. yfiK_rev**	TGAAATTCAGAGGCGGTAGAGTGACACCGACCCTTTTAAG
**3. pCysK_BB_fwd**	TCTACCGCCTCTGAATTTC
**4. pCysK_BB_rev**	TAATAAGCCCCGAGATGTC

Table A2. Metabolome data collected from the short-term experiments with E. coli W3110 pCysK. The presented metabolite concentrations are shown in units of mmol per unit of cell volume (mmol L^{-1}). Lower (grey) and upper (white) bounds for each metabolite concentration were calculated from the average and standard deviation of three LC-MS measurements for each sample. Metabolome data was meassured for each of the three feeding stages of the four parallel reactors fed with either glucose (G), pyruvate (P), a mixture of glucose and pyruvate (GP), or a mixture of glucse and succinate (GS as well as for the L-cysteine production process from which the cells for the short-term experiments were withdrawn (Ref).

Metabolite	Ref	G1	G2	G3	P1	P2	P3	GP1	GP2	GP3	GS1	GS2	GS3
13DPG	0.023	0.190	0.157	0.130	1.926	3.026	2.157	-	0.347	-	0.347	0.001	0.291
	0.023	0.190	0.157	0.130	1.926	3.026	2.157	-	0.347	-	0.347	0.001	0.291
2PG	0.004	0.005	0.005	0.005	0.022	0.033	0.023	1.385	0.001	1.385	1.761	0.031	0.005
	2.623	1.578	1.904	1.818	0.094	0.212	0.103	1.385	1.761	1.385	1.761	2.005	1.641
3PG	0.130	0.833	0.961	0.805	0.800	1.215	0.840	0.666	0.747	0.666	0.747	0.876	0.848
	0.130	0.833	0.961	0.805	1.211	2.276	1.859	0.666	0.747	0.666	0.747	0.876	0.848
3PHP	0.006	0.041	0.074	0.061	0.056	0.044	0.092	0.045	0.057	0.045	0.057	0.070	0.063
	0.006	0.041	0.074	0.061	0.056	0.044	0.092	0.045	0.057	0.045	0.057	0.070	0.063
6PGC	2.736	2.491	2.473	2.165	2.421	5.792	2.597	2.130	2.051	2.130	2.051	2.327	2.747
	3.185	3.277	3.249	2.825	3.169	7.583	3.431	2.908	2.824	2.908	2.824	3.214	3.518
AC	-	-	-	-	-	-	16.863	-	-	6.284	-	14.279	16.056
	-	-	-	-	-	-	37.529	-	-	39.508	-	60.636	49.106
AcCoA	1.958	2.131	2.556	2.222	2.111	4.592	2.208	1.881	2.283	1.881	2.283	2.761	2.077
	2.458	2.661	3.015	2.769	2.579	5.825	2.893	2.292	2.752	2.292	2.752	3.433	2.498
ADP	0.765	0.849	0.932	0.878	0.671	1.553	0.768	0.596	0.730	0.596	0.730	0.871	1.074
	1.200	1.337	1.469	0.878	1.095	2.640	0.768	1.069	1.135	1.069	1.135	1.393	1.481
AMP	0.084	0.319	0.534	0.301	0.246	0.310	0.420	0.315	0.304	0.315	0.304	0.637	0.540
	0.516	0.484	1.088	0.812	0.366	0.445	0.630	0.513	0.626	0.513	0.626	0.790	0.667
ATP	1.810	2.579	2.030	2.705	2.389	6.556	2.218	1.954	2.084	1.954	2.084	2.235	2.561
	2.426	3.402	3.284	2.705	3.388	6.556	2.828	2.608	2.874	2.608	2.874	3.114	3.122
DHAP	5.239	6.574	6.656	6.262	-	-	-	6.329	6.282	6.329	6.282	6.730	-
	5.239	6.574	6.656	6.262	-	-	-	6.329	6.282	6.329	6.282	6.730	-
E4P	9.983	3.022	16.107	24.898	-	-	-	19.486	18.887	19.486	18.887	22.431	-
	9.983	3.022	16.107	24.898	-	-	-	19.486	18.887	19.486	18.887	22.431	-
F6P	0.697	0.493	0.726	0.403	0.566	1.276	0.551	0.496	0.474	0.496	0.474	0.499	0.675
	1.047	1.190	1.296	1.011	0.899	1.943	0.967	0.831	0.889	0.831	0.889	0.906	1.040
FAD	0.158	0.242	0.249	0.209	0.247	0.532	0.262	0.213	0.208	0.213	0.208	0.237	0.272
	0.207	0.316	0.325	0.273	0.318	0.699	0.343	0.286	0.287	0.286	0.287	0.319	0.343
FDP	0.152	0.369	0.388	0.343	0.376	0.778	0.407	0.329	0.328	0.329	0.328	0.404	0.412
	0.313	0.492	0.505	0.447	0.514	1.057	0.548	0.456	0.449	0.456	0.449	0.516	0.535
G6P	0.284	0.261	0.474	0.459	0.210	0.338	0.293	0.279	0.445	0.279	0.445	0.795	0.262
	0.644	0.358	0.594	0.709	0.269	0.438	0.394	0.436	0.568	0.436	0.568	1.198	0.347
MAL	0.087	0.085	0.055	0.056	-	-	-	-	-	-	-	-	-
	0.087	0.085	0.055	0.056	-	-	-	-	-	-	-	-	-
NAD	1.059	0.779	0.811	0.708	0.566	0.779	0.761	0.628	0.751	0.628	0.751	0.798	0.983
	1.059	1.137	1.310	1.149	0.878	1.047	0.976	1.051	1.112	1.051	1.112	1.360	1.166
NADH	0.303	0.319	0.238	0.212	0.264	0.751	0.275	0.215	0.203	0.215	0.203	0.238	0.218
	0.459	0.469	0.455	0.385	0.420	1.201	0.473	0.395	0.351	0.395	0.351	0.465	0.413

Table A2. Continuation

Metabolite	Ref	G1	G2	G3	P1	P2	P3	GP1	GP2	GP3	GS1	GS2	GS3
**NADP**	0.416	0.390	0.382	0.336	0.357	0.736	0.383	0.341	0.337	0.341	0.337	0.395	0.448
	0.463	0.505	0.490	0.437	0.422	0.946	0.494	0.447	0.452	0.447	0.452	0.519	0.534
**NADPH**	0.767	1.364	1.281	0.988	1.260	2.551	1.507	1.210	1.239	1.210	1.239	1.445	1.582
	1.031	1.624	1.910	1.262	1.617	3.467	1.832	1.477	1.589	1.477	1.589	1.821	1.870
**PEP**	0.281	0.311	0.344	0.285	0.291	0.624	0.332	0.276	0.654	0.276	0.654	0.296	0.308
	0.471	0.464	0.473	0.454	0.429	0.964	0.468	0.447	0.446	0.447	0.446	0.515	0.438
**PSER__L**	12.029	6.945	11.040	12.165	4.870	6.177	0.332	6.675	9.902	6.675	9.902	15.620	9.582
	28.021	8.475	12.998	15.016	6.498	9.492	0.468	8.046	13.385	8.046	13.385	22.569	12.593
**PYR**	0.933	1.651	1.015	0.112	-	0.352	-	-	-	-	-	2.103	1.611
	0.933	1.651	1.015	0.817	-	4.776	-	-	-	-	-	14.161	1.611
**S7P**	0.195	0.190	0.201	0.172	0.197	0.439	0.186	0.158	0.150	0.158	0.150	0.189	0.205
	0.212	0.273	0.280	0.256	0.286	0.602	0.277	0.243	0.227	0.243	0.227	0.265	0.283
**SCYS__L**	9.799	2.758	2.874	3.953	1.698	4.637	3.162	3.157	3.166	3.157	3.166	4.742	4.787
	35.840	9.017	13.343	11.565	7.926	12.656	13.851	8.517	11.463	8.517	11.463	10.739	13.955

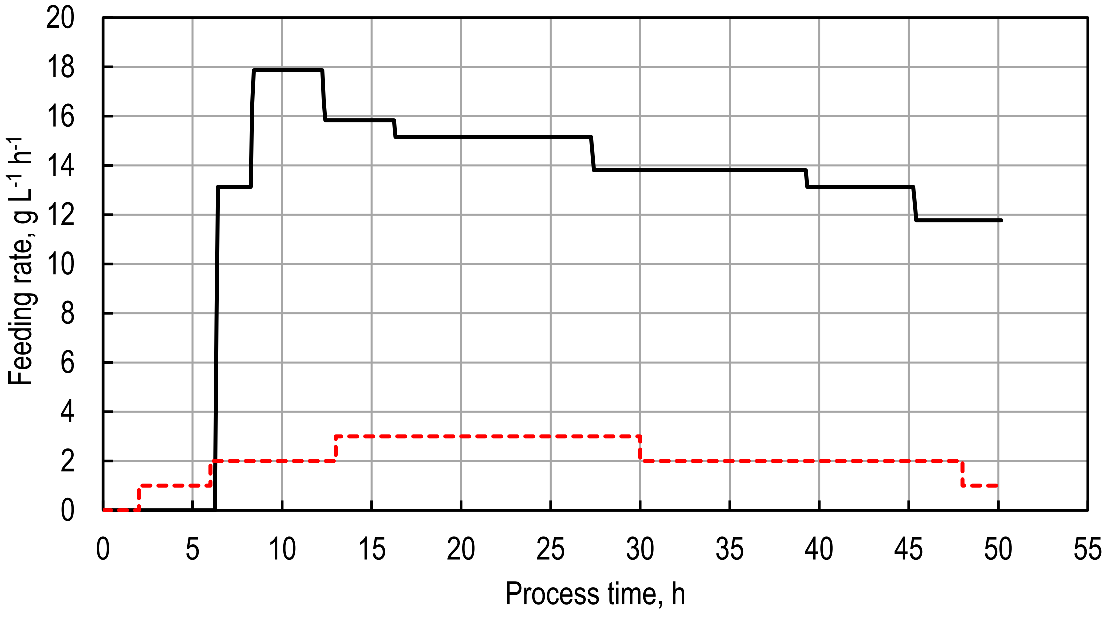

Figure A1. Substrate feeding rates from the fed-batch L-cysteine production process. The D-glucose (*continuous line*) and ammonium thiosulfate (*broken line*) feeding rates are presented in grams per unit of initial reactor volume per unit of time.

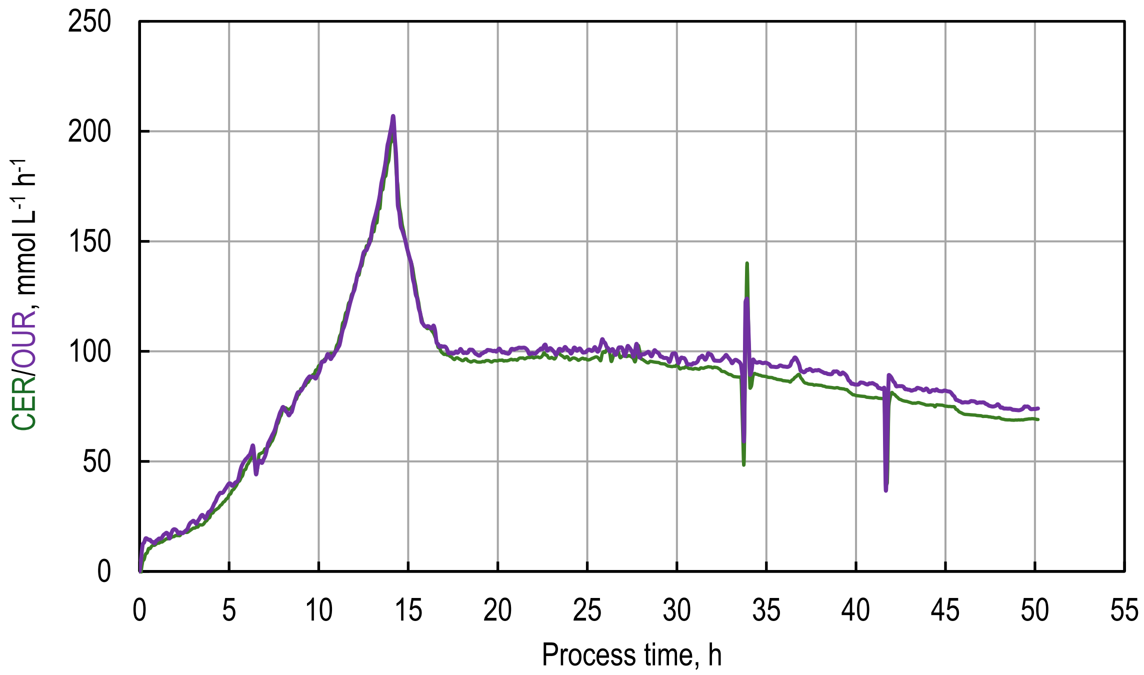

Figure A2. Gas exchange rates of the fed-batch L-cysteine production process with E. coli W3110 pCysK. The carbon evolution (CER, green) and oxygen uptake rates (OUR, purple) were calculated from the CO_2 and O_2 contents measured at the gas exhaust of the stirred tank reactor respectively.

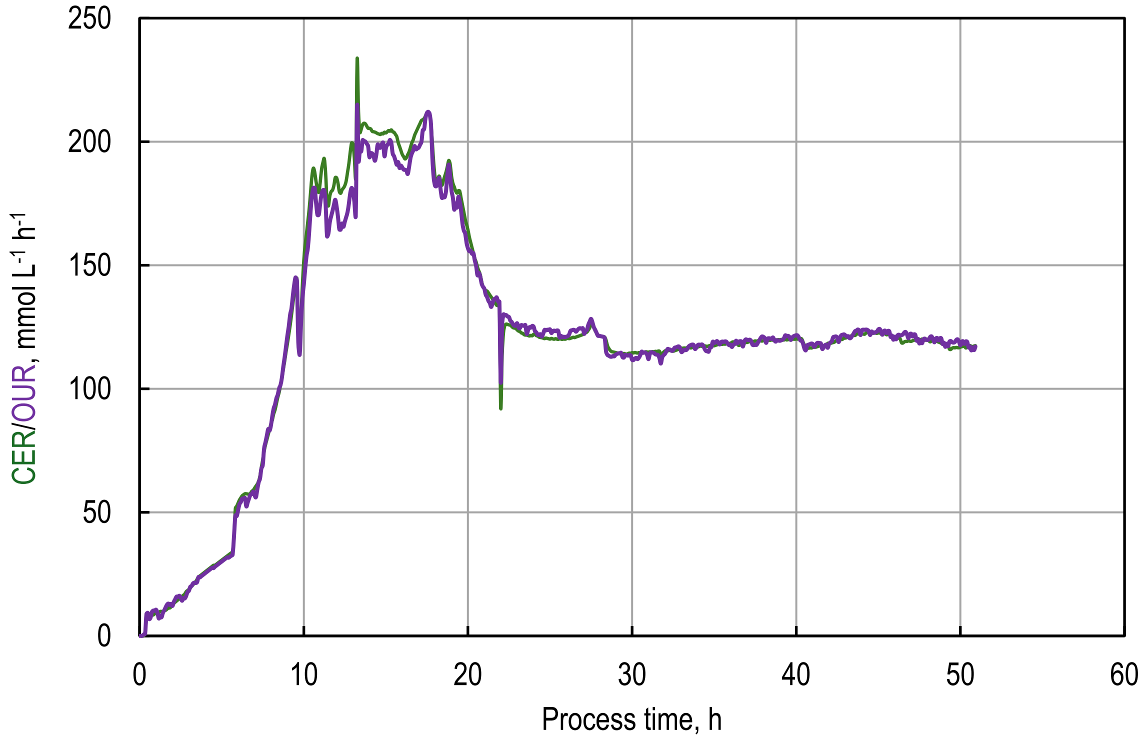

Figure A3. Gas exchange rates of the fed-batch L-cysteine production process with E. coli W3110 pCysK_yfiK. The carbon evolution (CER, green) and oxygen uptake rates (OUR, purple) were calculated from the CO_2 and O_2 contents measured at the gas exhaust of the stirred tank reactor respectively. The profiles correspond to the average CER and OUR values of three individual L-cysteine production processes.

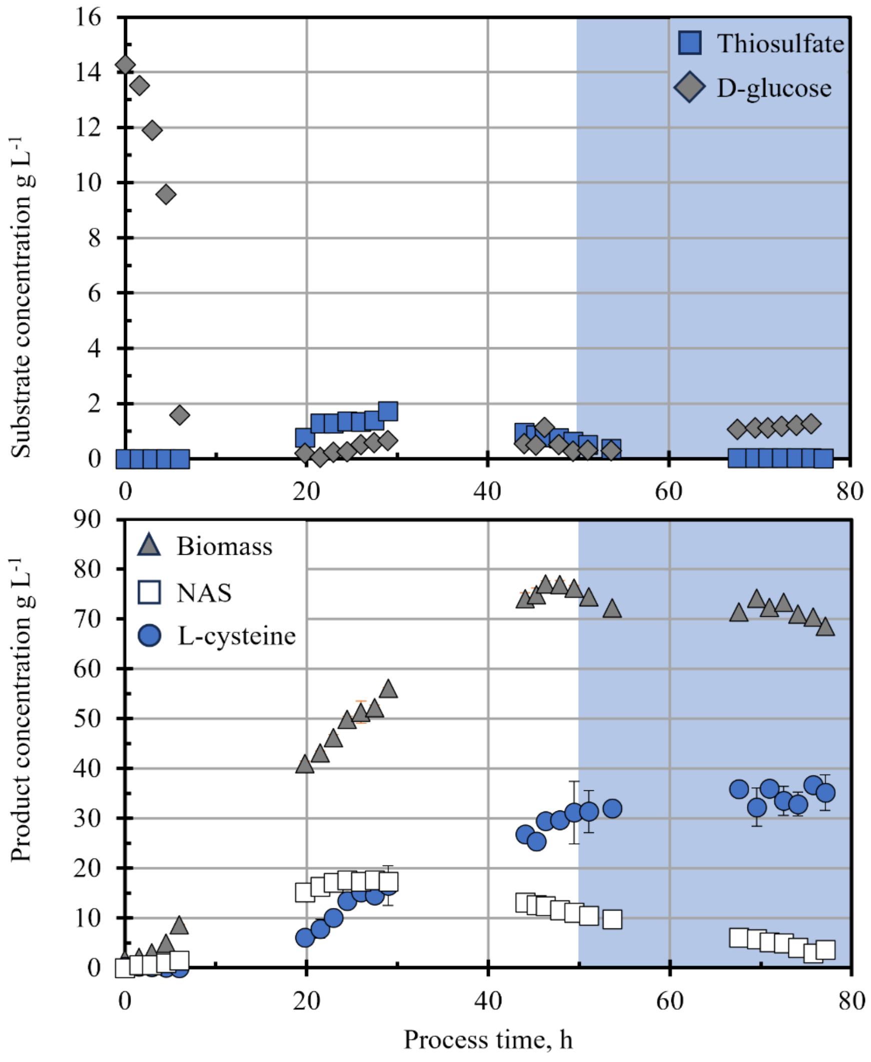

Figure A4. Extended fed-batch L-cysteine production process with E. coli W3110 pCysK_yfiK_nRBS. The cultivation time of this experiment was extended from 50 to 78 hours to characterize the extent of the prolonged L-cysteine productivity observed as a result of the L-cysteine exporter exchange. The shaded area indicates the time extension in comparison to the standard fed-batch production process time of 50 hours.
